# Using Virtual Reality to Enhance Surgical Skills and Engagement in Orthopedic Education: Systematic Review and Meta-Analysis

**DOI:** 10.2196/70266

**Published:** 2025-05-30

**Authors:** Ting Li, Jingxin Yan, Xin Gao, Hangyu Liu, Jin Li, Yuanting Shang, Xiaoyu Tang

**Affiliations:** 1 Department of Orthopedics, No. 1 Orthopedics Hospital of Chengdu, 389 Jinhui Road, Chengdu, 610031, China, 86 02886635247; 2 School of Medicine, South China University of Technology, Guangzhou, China; 3 Department of Orthopedics, Sichuan Provincial People's Hospital, School of Medicine, University of Electronic Science and Technology of China, Chengdu, China

**Keywords:** meta-analysis, virtual reality, traditional education, randomized controlled trial, VR

## Abstract

**Background:**

Currently, virtual reality (VR) simulators are of increasing interest for surgical training, but there is no systematic review exploring the advantages and disadvantages of VR in orthopedic education.

**Objective:**

This paper aims to explore the relationship between VR education and traditional education.

**Methods:**

We searched PubMed, Embase, Web of Science, Cochrane library, Scopus, Chongqing VIP Database (VIP), Chinese National Knowledge Infrastructure (CNKI), and Wan Fang Database up to July 2024 for relevant studies. A total of 2 investigators independently conducted literature screening, data extraction, and risk of bias assessment for included studies in accordance with the PICOS framework (Population, Intervention, Comparison, Outcomes, and Study Design), followed by statistical synthesis of outcomes using RevMan 5.3 software (Cochrane Collaboration). The risk of bias evaluation adhered to the Cochrane Risk of Bias Tool (RoB 2.0) for randomized controlled trials, ensuring systematic appraisal of sequence generation, allocation concealment, blinding, incomplete outcome data, and selective reporting.

**Results:**

A total of 23 randomized controlled trials included 1091 participants in this meta-analysis. The majority of studies focused on the undergraduates (n=3) and trainees (n=8), resident doctors (n=10), and postgraduate doctors (n=2). A total of 3 studies were missing the age of participants, and 5 studies were also missing the duration data. The main outcome included knowledge scores, clinical operation scores, surgical design scores, and so on. The secondary outcomes were included course participation, learning efficiency, enhance clinical ability, and so on. Compared to traditional teaching, VR interventions resulted in significantly higher knowledge scores (standardized mean difference [SMD]=1.08, 95% CI 0.71-1.46; *P*<.001). Furthermore, VR-based education yielded superior clinical operation scores (SMD=1.44, 95% CI 1.07-1.81; *P*<.001) and surgical design scores (SMD=1.75, 95% CI 1.05-2.44; *P*<.001). In addition, VR teaching enhanced clinical understanding (SMD=1.05, 95% CI 0.62-1.48; *P*<.001) and clinical thinking ability (SMD=1.17, 95% CI 0.66-1.68; *P*<.001) compared to traditional methods. Furthermore, VR teaching was associated with higher levels of teaching interest (odds ratio [OR]=4.17, 95% CI 2.16-8.04; *P*<.001) and teaching satisfaction (OR 4.13, 95% CI 1.96-8.69; *P*<.001) than traditional approaches. Finally, VR significantly enhanced the initiation of learning among students when compared with traditional teaching methods (SMD=1.15, 95% CI 0.91-1.39; *P*<.001).

**Conclusions:**

This meta-analysis emphasizes VR as an excellent orthopedic educational tool. It significantly enhances both theoretical knowledge and practical skills, while also markedly increasing student engagement and satisfaction. Therefore, adopting VR technology in medical education holds promise for improving orthopedic surgical competence. However, the quality of this meta-analysis was limited by the notable heterogeneity in terms of VR platforms these findings and further validation through multicenter, double-blind, and large-sample randomized controlled trials is required.

## Introduction

The traditional education model has historically played a crucial role in knowledge dissemination and skill development. However, it faces several challenges, including enhancing engagement in learning experiences and effectively addressing individualized learning needs of students [1,2]. In this context, virtual reality (VR) teaching has emerged as a groundbreaking tool with profound implications for medical education and clinical practice across various specialties, including orthopedics [3]. VR’s ability to create immersive, interactive, and lifelike simulated environments offers unique advantages in training and enhancing the capabilities of orthopedic surgeons and medical students [4,5].

Orthopedic surgery necessitates a thorough grasp of intricate anatomical structures and precise surgical techniques. Nevertheless, conventional educational approaches primarily involve cadaveric dissection, operating room observations, and simulation models, which may offer limited benefits in augmenting the academic performance and clinical aptitude of orthopedic surgeons [6,7]. In contrast, VR simulations have the capacity to replicate detailed anatomical structures and surgical procedures accurately, enabling learners to interact with and manipulate virtual models in real time. This capability significantly enhances the assimilation of theoretical knowledge and the refinement of practical skills [8,9]. However, VR teaching encounters several challenges. First, there is the issue of technological expense, as procuring equipment and software can impose financial burdens on educational institutions. Second, the fidelity of virtual environments remains constrained, often hindering practitioners from experiencing the genuine sensations of real surgeries within virtual settings [10].

Hence, there is still some controversy over whether VR teaching is appropriate in orthopedic education. This paper aims to explore the relationship between VR education and traditional education, as well as their interactive influences. Through meta-analysis and literature review, we will examine the advantages, challenges, and potential of VR education from multiple perspectives.

## Methods

### Study Design

This a systematic review and meta-analysis is in accordance with PRISMA (Preferred Reporting Items for Systematic Reviews and Meta-Analyses; see Checklist 1) statement and AMSTAR (Assessing the Methodological Quality of Systematic Reviews) guidelines, and it was conducted following the guidelines of the Cochrane Handbook [11]. It is registered in PROSPERO (International Prospective Register of Systematic Reviews; CRD42024592192).

### Literature Retrieval Strategy

The following electronic databases were searched up to July 2024, such as PubMed, Embase, Cochrane library, Web of Science, Scopus, CNKI, VIP, and Wan Fang Database. All randomized controlled trials (RCTs) comparing VR teaching with traditional teaching were collected. The following keywords were used: “virtual reality” OR VR OR “augmented reality” OR “simulation”) AND (“orthopedics” OR “orthopedic education”). These keywords were used as MeSH (Medical Subject Headings) headings and free text words. Additional searches of relevant references within included articles and existing systematic reviews were performed manually. The specific search strategy for the electronic database is described in Multimedia Appendix 1.

### Inclusion and Exclusion Criteria

#### Inclusion Criteria

Studies were eligible for inclusion if they met the following criteria of PICOS (Population, Intervention, Comparison, Outcomes, and Study Design): (1) population: target population is medical students; (2) intervention: VR teaching; (3) comparison: traditional teaching; (4) outcomes: knowledge scores, clinical operation scores, surgical design scores, clinical understanding ability, clinical thinking ability, initiation of learning, teaching interest, teaching satisfaction, and so on; and (5) study design: all RCTs were included.

#### Exclusion Criteria

Studies were ineligible if they met the following criteria of PICOS: (1) population: studies involving nonmedical student populations; (2) intervention: studies not using VR as a primary teaching modality; (3) comparison: studies lacking a direct comparison to traditional teaching methods were excluded; (4) outcomes: studies failing to report at least one predefined outcome measure (knowledge scores, clinical operation design scores, clinical understanding ability, clinical thinking ability, learning engagement, teaching interest, teaching satisfaction, and so on) were excluded; and (5) study design: non-RCTs (eg, observational studies, case reports, and cohort studies) were excluded.

### Data Extraction

The process was carried out independently by 2 reviewers following the Cochrane Collaboration guidelines for systematic reviews. In addition, 2 researchers independently reviewed the full texts of potentially eligible studies based on predefined inclusion and exclusion criteria. The data were extracted as follows: (1) trial name (author and year of publication), study design; (2) population characteristics, including, sample size, age, and teaching subjects; and (3) type of intervention and intervention duration. Disagreements on eligibility were first resolved by discussion and decided by a third reviewer if disagreement persisted.

The study outcomes were quantified through multidimensional assessments, including (1) knowledge scores: knowledge scores evaluated theoretical mastery using change between pre- and posttest scores; (2) clinical operation scores: a composite metric quantifying technical proficiency in clinical procedures, emphasizing procedural standardization, anatomical accuracy, and operative safety; (3) operation design scores: an evaluation of surgical plan rationality and innovation, focusing on process design (eg, incision selection, instrument configuration, and procedural sequence optimization) to reflect preoperative strategic logic; (4) clinical understanding ability: a measure of integration between theoretical knowledge and clinical practice, encompassing disease mechanism analysis, anatomical structure comprehension, and treatment plan coherence; (5) clinical thinking ability: an assessment of analytical reasoning, decision-making, and prioritization in clinical scenarios, including information synthesis, differential diagnosis logic, and therapeutic intervention hierarchies; (6) initiative ability: a metric evaluating proactive problem identification, self-driven learning, and anticipatory risk mitigation, such as autonomous learning path design, workflow optimization, and preemptive clinical risk resolution; (7) teaching interest: an evaluation of curriculum engagement, interactive facilitation capabilities, and responsiveness to learner feedback; and (8) teaching satisfaction: a subjective measure of satisfaction with instructional content, methodology, and efficacy, typically assessed via surveys, post-course ratings, or longitudinal participation intent.

We also extracted data on secondary outcomes, including (1) course participation: attendance, task completion rates, and active engagement levels during training; (2) learning efficiency: the ratio of knowledge and skill acquisition velocity to resource expenditure, for example, VR versus traditional group accuracy improvements per unit time; (3) enhance clinical ability: posttraining skill advancement (eg, diagnostic precision and operative proficiency), validated via pre-post simulated and real-world performance comparisons; (4) novelty of teaching: innovation in pedagogical methods (eg, VR integration and gamification), evaluated through learner feedback or comparative efficacy against conventional approaches; (5) solve problem ability: logical rigor and efficacy in formulating solutions for complex clinical scenarios (eg, rare complications and multidisciplinary cases); (6) interactive ability: competence in team collaboration, clinician-student communication, and patient interactions, including clarity of expression, active listening, and collaborative problem resolution; (7) self-study ability: capacity for autonomous learning goal-setting, resource curation, and knowledge assimilation, measured via post-course literature review completion or independent competency assessments; (8) self-confidence: confidence in technical skills and clinical decisions; and (9) train time: duration required to achieve predefined competency benchmarks or curriculum cycle optimization.

### Quality Assessment

The risk of bias assessment was carried out by 2 independent researchers using the Cochrane Risk of Bias tool 2.0 [12]. Any unresolved disagreements between reviewers were resolved through discussion or by evaluation by a third reviewer. The methodological quality of the studies was evaluated using the Cochrane Risk of Bias tool 2.0, which assesses randomization process, deviation from intended interventions, missing outcome data, measurement of the outcome, and selection of the reported result. In addition, every item was rated as “low risk of bias,” “unclear risk of bias,” or “high risk of bias.”

### Statistical Analysis

All data were analyzed using Revman 5.3 software. The dichotomous outcomes were reported by odds ratio (OR) with 95% CI and we reported continuous outcomes for standardized mean difference (SMD) with 95% CI. Heterogeneity was evaluated with *I*^2^ and *P* values. If *I^2^*≤50%, it indicated that there was no homogeneity among the research results, and a fixed effect model was used. If *P*<.05 and *I^2^*>50%, then, heterogeneity existed among studies, and a random effect model was used. Publication bias was examined using funnel plots. Statistical significance was defined as *P*<.05 for all analyses.

The assessment of clinical outcomes was rigorously conducted in strict adherence to the Grading of Recommendations Assessment, Development, and Evaluation (GRADE) guidelines. Given that all included studies were RCTs, predefined downgrading criteria were systematically applied: a 1-point downgrade was implemented when the 95% CI of risk ratios crossed the null value. In pooled analyses, additional downgrades for imprecision were applied to very small sample sizes, with a “serious” quality reduction assigned to study arms with <50 participants. The GRADE quality evaluation was performed independently by 2 reviewers, and any discrepancies were resolved through iterative discussion and consensus-based adjudication.

## Results

### Search Result

**Figure 1.**
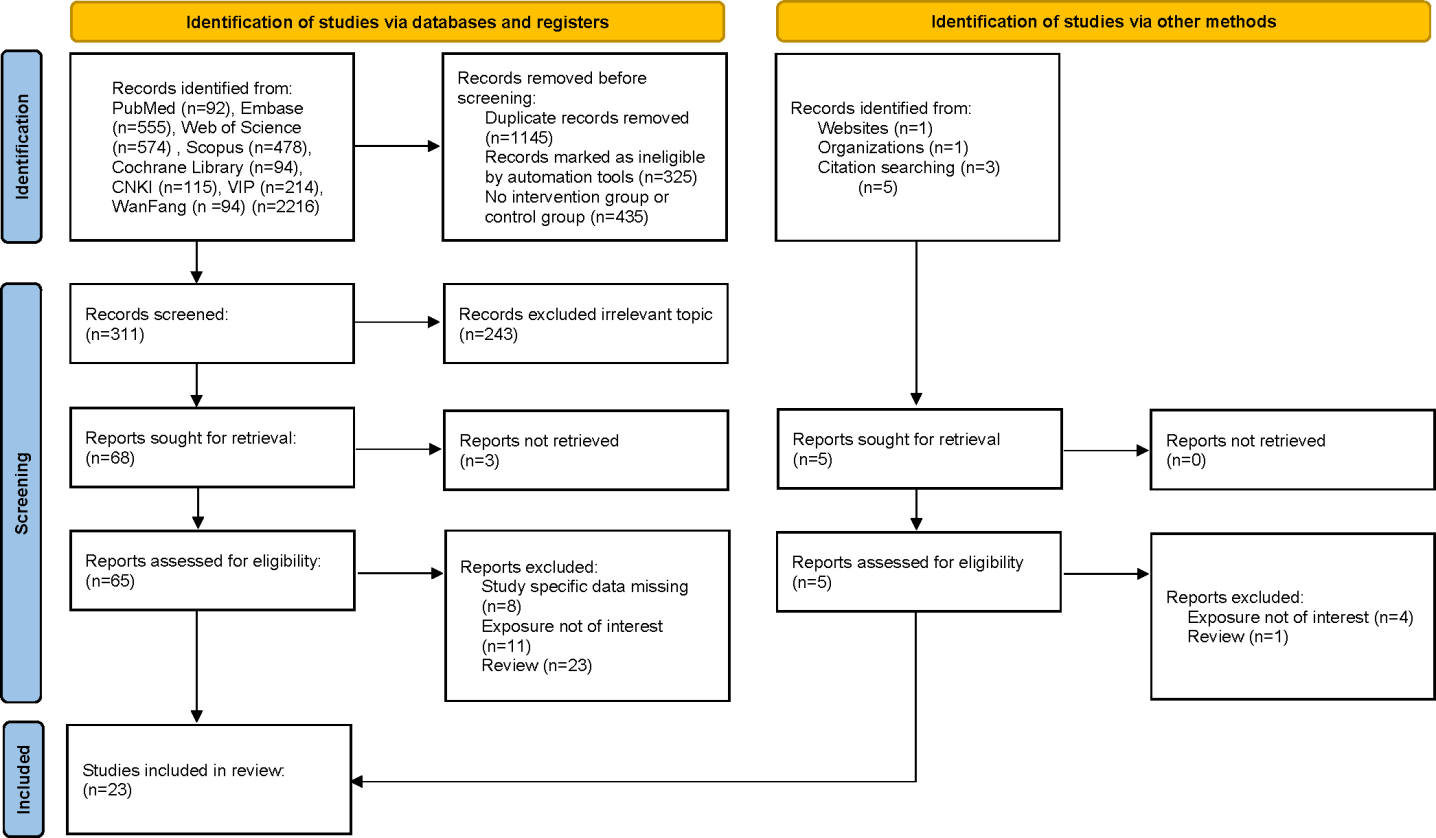
Study design flowchart.

The initial search yielded 2221 records up to July 2024, where we excluded 1145 records due to the duplication. After examination of the titles, abstracts, and full-text articles, 23 [13-35] potentially eligible studies assessed for inclusion criteria, 3 trials published in English and 20 trials published in Chinese were included in this meta-analysis. The publication years of the included studies were from 2014 to 2023. The studies with a sample size between 18 and 125 participants were included. Figure 1 shows the selection algorithm and the numbers of included and excluded studies. All titles, abstracts, and text were dually and independently reviewed by the authors based on the inclusion and exclusion criteria to minimize bias.

### Study Characteristics

A total of 3 RCTs included 1091 participants in this meta-analysis. The majority of studies focused on the undergraduates (n=3) and 8 studies for trainees, 10 for resident doctors, and 2 for postgraduate doctors. A total of 3 studies were missing the age of participants and 5 studies were also missing the duration data. Table 1 shows the main basic characteristics of the included studies.

**Table 1.** Basic characteristics of the included studies.

Study	Year	Study type	Population	Mean age (years)		Persons, n		Intervention group	Control group	Teaching subjects	Study duration	
				I^a^	C^b^	I	C				I	C
Gao et al [15]	2019	RCT^c^	Trainees	25.6	26.2	21	21	VR^d^	Traditional teaching	The correction of severe anomalies of lower extremity	7 months	7 months
Ding et al [14]	2021	RCT	Undergraduates	21.16	21.32	25	25	VR+PBL^e^	Traditional teaching	N/A^f^	N/A	N/A
Hao et al [16]	2021	RCT	Trainees	23.1	23.1	12	12	VR	Traditional teaching	Sacral fracture fixation	6 months	6 months
Huang et al [17]	2021	RCT	Resident doctors	24.2	24.4	18	18	VR	Traditional teaching	Ankle and femoral fracture	3 months	3 months
Li et al [18]	2023	RCT	Resident doctors	22.4	22.3	40	40	VR+PBL	Traditional teaching	Trauma orthopedics	24 months	24 months
Li et al [19]	2021	RCT	Resident doctors	25.44	25.69	32	32	VR	Traditional teaching	N/A	6 months	6 months
Li et al [20]	2023	RCT	Nursing trainees	21.78	21.54	30	30	VR	Traditional teaching	Orthopedics nursing	5 months	6 months
Liu et al [21]	2023	RCT	Resident doctors	07	24.93	30	30	VR	Traditional teaching	Fracture of distal radius	18.5 months	18.5 months
Liu et al [22]	2019	RCT	Trainees	22.3	22.4	20	20	VR+CBL^g^	Traditional teaching	N/A	2 months	2 months
Ma and Wu [24]	2023	RCT	Resident doctors	27.50	27.60	20	20	VR+CBL	Traditional teaching	Knee arthroscopic	14 months	14 months
Meng et al [26]	2018	RCT	Trainees	23.17	23.09	35	35	VR	Traditional teaching	Fractures	24 months	24 months
Nie et al [27]	2023	RCT	Undergraduates	22.50	23.24	20	20	VR+PBL	Traditional teaching	Knee arthroscopic	13 months	13 months
Tao et al [28]	2023	RCT	Resident doctors	25.13	25.03	40	40	VR	Traditional teaching	Spinal cord injury	32 months	32 months
Wang et al [29]	2014	RCT	Postgraduate doctors	35.42	34.01	20	16	VR	Traditional teaching	Knee arthroscopic	1.5 months	1.5 months
Wei et al [30]	2023	RCT	Trainees	22.3	22.3	65	60	VR+PBL	Traditional teaching	Fracture of distal radius	21 months	21 months
Yu et al [31]	2019	RCT	Trainees	N/A	N/A	12	12	VR	Traditional teaching	Spine	N/A	N/A
Zhang et al [32]	2022	RCT	Resident doctors	24.17	24.61	18	18	VR	Traditional teaching	Trauma orthopedics	5 months	5 months
Zhu et al [33]	2022	RCT	Postgraduate doctors	96	24.96	12	12	VR	Traditional teaching	N/A	N/A	N/A
Zou et al [34]	2022	RCT	Trainees	25.2	25.3	30	30	VR	Traditional teaching	Knee arthroscopy	3 months	3 months
Zuo et al [35]	2020	RCT	Undergraduates	21.22	21.55	20	20	VR	Traditional teaching	N/A	N/A	N/A
McKinney et al [25]	2022	RCT	Resident doctors	N/A	N/A	11	11	VR	Traditional teaching	Knee arthroplasty	N/A	N/A
Blumstein et al [13]	2020	RCT	Resident doctors	N/A	N/A	10	10	VR	Traditional teaching	N/A	0.5 months	0.5 months
Lohre et al [23]	2020	RCT	Resident doctors	31.1	31.0	9	9	VR	Traditional teaching	Orthopedic surgical skills	3 days	3 days

a I: intervention.

b C: comparison.

c RCT: randomized controlled trial.

d VR: virtual reality.

e PBL: problem-based learning.

f N/A: not available.

g CBL: case-based teaching.

### The Bias Risk Assessment Results of the Included Studies

**Figure 2.**
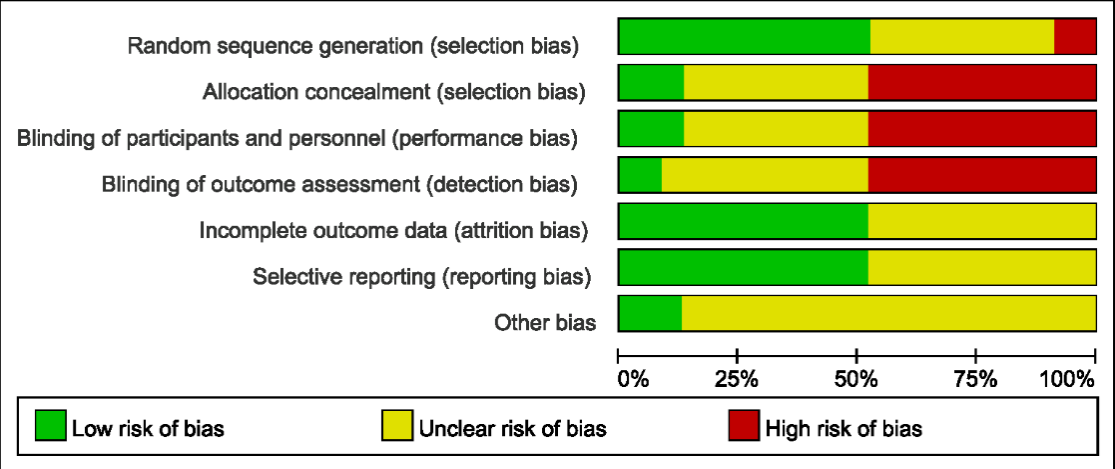
Results of quality assessment using the Cochrane risk tool.

The risk of bias of RCTs were evaluated by the Cochrane tool. The authors showed the results of each quality item as percentages across studies. Two studies did not report the RCT design, 9 studies are ambiguous about random sequence generation, and 12 studies claimed the RCT design. It was found that 3 studies were of low-risk bias and therefore had some concerns about the risk of bias for many of the criteria. The quality assessment of included studies was shown in Figure 2 for details. Outcome-level quality assessment was conducted using the GRADE methodology, with comprehensive documentation provided in Multimedia Appendix 2. The overall certainty of evidence, evaluated in accordance with GRADE criteria, was categorized as moderate to very low.

### Primary Meta-Analysis Results

#### Knowledge Scores

**Figure 3.**
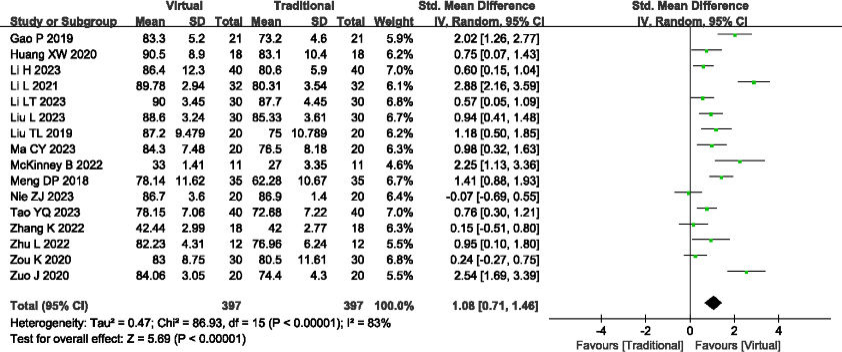
A forest plot showing the knowledge scores [15,17-22,24-28,32-35].

A total of 16 studies (n=794) [15,17-22,24-28,32-35] reported the knowledge scores. Significant heterogeneity was observed (*P*<.001; *I^2^*=83%), necessitating the use of a random effects model. VR teaching demonstrated significantly higher knowledge scores compared to traditional teaching methods (SMD=1.08, 95% Cl 0.71-1.46; *P*<.001; see Figure 3). Sensitivity analysis was conducted to identify potential sources of heterogeneity, but no significant source was identified. Outcome level quality for knowledge scores assessed by GRADE was “very low.”

#### Clinical Operation Scores

**Figure 4.**
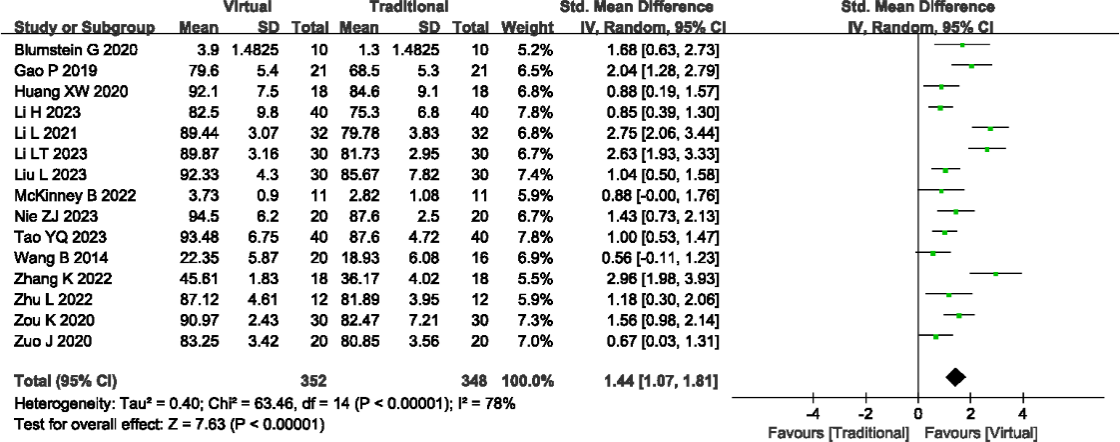
A forest plot showing the clinical operation scores [13,15,17-21,25,27-29,32-35]

A total of 15 studies (n=700) [13,15,17-21,25,27-29,32-35] reported the clinical operation scores. Significant heterogeneity among studies was observed, prompting the use of a random effects model (*P*<.001; *I^2^*=78%). VR teaching demonstrated significantly higher clinical operation scores compared to traditional teaching methods (SMD=1.44, 95% Cl 1.07-1.81; *P*<.001; see Figure 4). Sensitivity analysis was conducted to investigate potential sources of heterogeneity, revealing no significant sources. Outcome level quality for clinical operation scores assessed by GRADE was “very low.”

#### Operative Design Scores

**Figure 5.**
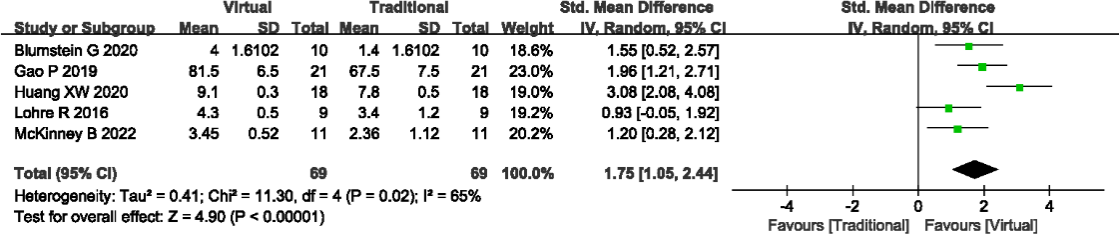
A forest plot showing the operative design scores [13,15,17,23,25].

A total of 5 studies (n=138) [13,15,17,23,25] reported the operation design scores. Significant heterogeneity was observed among the studies (*P*=.02; *I^2^*=65%), necessitating the use of a random-effects model. Meta-analysis indicated that VR-based teaching methods yielded significantly higher operative design scores compared to traditional teaching methods (SMD=1.75, 95% CI 1.05-2.44; *P*<.001; see Figure 5). Sensitivity analysis was conducted to explore potential sources of heterogeneity, yet no significant contributing factors were identified. Outcome level quality for operative design scores assessed by GRADE was “very low.”

#### Clinical Understanding Ability

**Figure 6.**
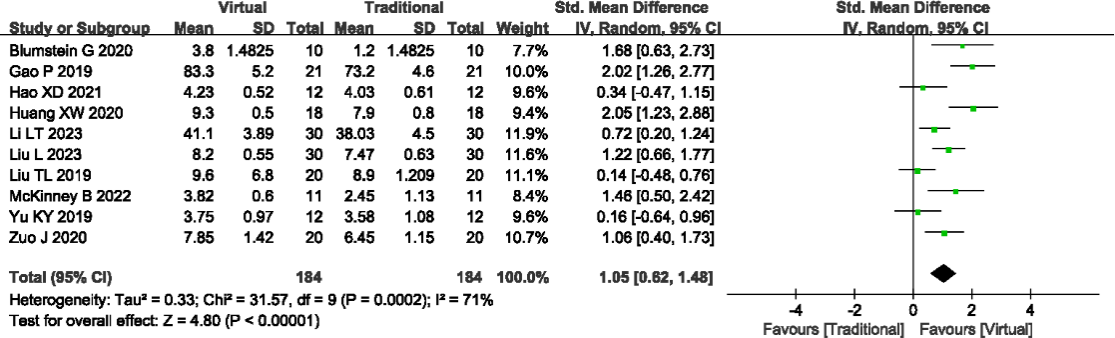
A forest plot showing the clinical understanding ability [13,15-17,20-22,25,31,35].

A total of 10 [13,15-17,20-22,25,31,35] studies (n=368) reported the clinical understanding ability. Significant heterogeneity was observed among the studies (*P*<.001, *I^2^*=71%), necessitating the utilization of a random-effects model. Meta-analysis revealed that VR-based teaching methods were associated with significantly higher clinical understanding ability compared to traditional teaching methods (SMD=1.05, 95% CI 0.62-1.48, *P*<.001; Figure 6). Sensitivity analysis was conducted to explore potential sources of heterogeneity, yet no significant contributing factors were identified. Outcome level quality for clinical understanding ability assessed by GRADE was “very low.”

#### Clinical Thinking Ability

**Figure 7.**
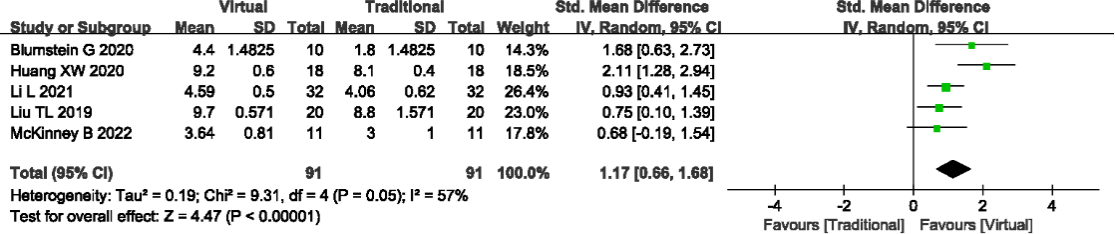
A forest plot showing the clinical thinking ability [13,17,19,22,25].

A total of 5 studies (n=182) [13,17,19,22,25] reported the clinical thinking ability. Significant heterogeneity was noted among the studies (*P*=.05; *I^2^*=57%), prompting the adoption of a random-effects model. The meta-analysis demonstrated that VR-based teaching methods were associated with significantly higher clinical thinking ability compared to traditional teaching methods (SMD=1.17, 95% Cl 0.66-1.68; *P*<.001; see Figure 7). Sensitivity analysis was conducted to explore potential sources of heterogeneity; however, no significant contributors were identified. Outcome level quality for clinical thinking ability assessed by GRADE was “very low.”

#### Teaching Interest

**Figure 8.**
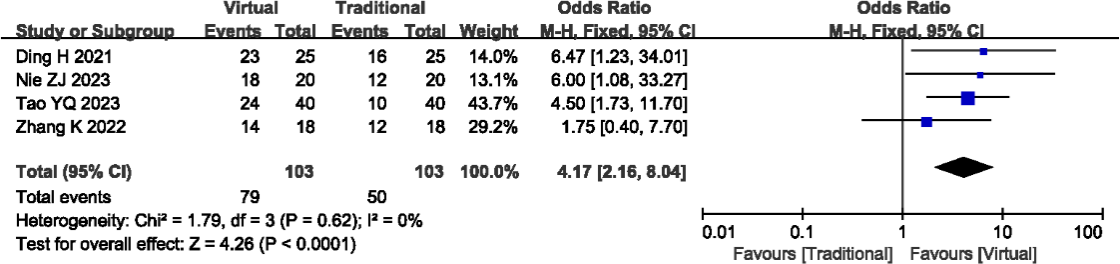
A forest plot showing the teaching interest [14,27,28,32].

A total of 4 studies (n=206) [14,27,28,32] reported the teaching interest. There was no significant heterogeneity observed among the studies (*P*=.62; *I^2^*=0%), thus a fixed-effects model was used. Our meta-analysis revealed that VR-based teaching methods were associated with significantly higher teaching interest compared to traditional teaching methods (OR 4.17, 95% Cl 2.16-8.04; *P*<.001; see Figure 8). Outcome level quality for teaching interest assessed by GRADE was “low.”

#### Teaching Satisfaction

**Figure 9.**
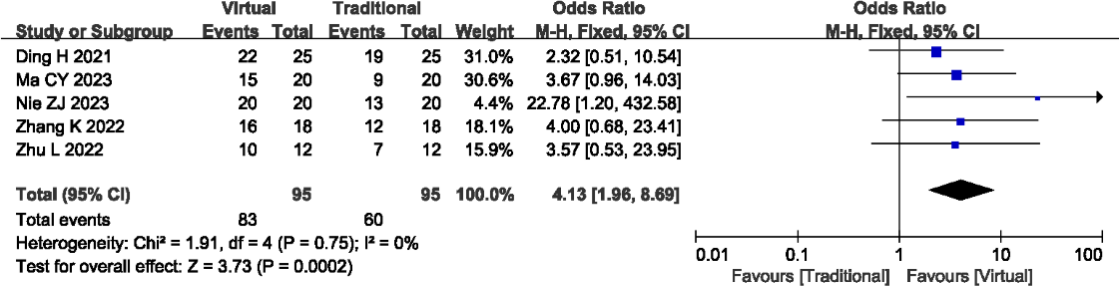
A forest plot showing the teaching satisfaction [14,24,27,32,33].

A total of 5 studies (n=190) [14,24,27,32,33] reported the teaching satisfaction. Statistical analysis indicated no significant heterogeneity among the studies (*P*=.75; *I^2^*=0%), thus a fixed-effects model was applied. The findings revealed significantly higher levels of teaching satisfaction associated with VR-based teaching (OR 4.13, 95% CI 1.96-8.69; *P*<.001; Figure 9). Outcome level quality for teaching satisfaction assessed by GRADE was “low.”

#### Initiative Ability

**Figure 10.**
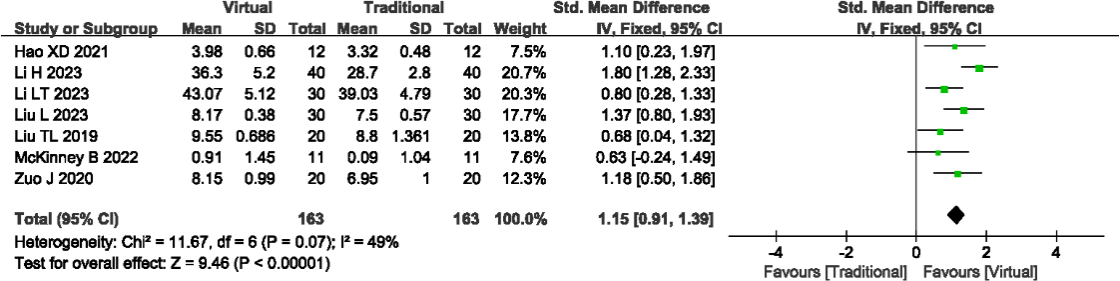
A forest plot showing the initiative ability [16,18,20-22,25,35].

A total of 7 studies (n=326) [16,18,20-22,25,35] reported the initiative ability. The analysis revealed nonsignificant heterogeneity across studies (*P*=.07; *I^2^*=49%), thus a fixed-effects model was used. Results demonstrated that VR-based teaching yielded significantly higher levels of initiative ability compared to traditional teaching methods (SMD=1.15, 95% Cl 0.91-1.39; *P*<.001; see Figure 10). Outcome level quality for initiative ability assessed by GRADE was “moderate.”

### Secondary Meta-Analysis Results

Our meta-analysis examined various clinical outcomes comparing VR teaching with traditional methods. VR teaching demonstrated superiority over traditional teaching in several domains: course participation (Figure S1 in Multimedia Appendix 3). VR teaching in the learning efficiency, enhance clinical ability, novelty of teaching, and solve problem ability were higher (Figures S2-S5 Multimedia Appendix 3). Furthermore, we also found that VR teaching was higher in the interactive ability, self-study ability, and self-confidence than traditional teaching (Figures S6-S8 in Multimedia Appendix 3). Finally, we also found that VR teaching was lower in the train time than traditional teaching (Figure S9 in Multimedia Appendix 3). Outcome level quality for secondary meta-analysis results assessed by GRADE was “moderate” to “very low.”

### Publication Bias

**Figure 11.**
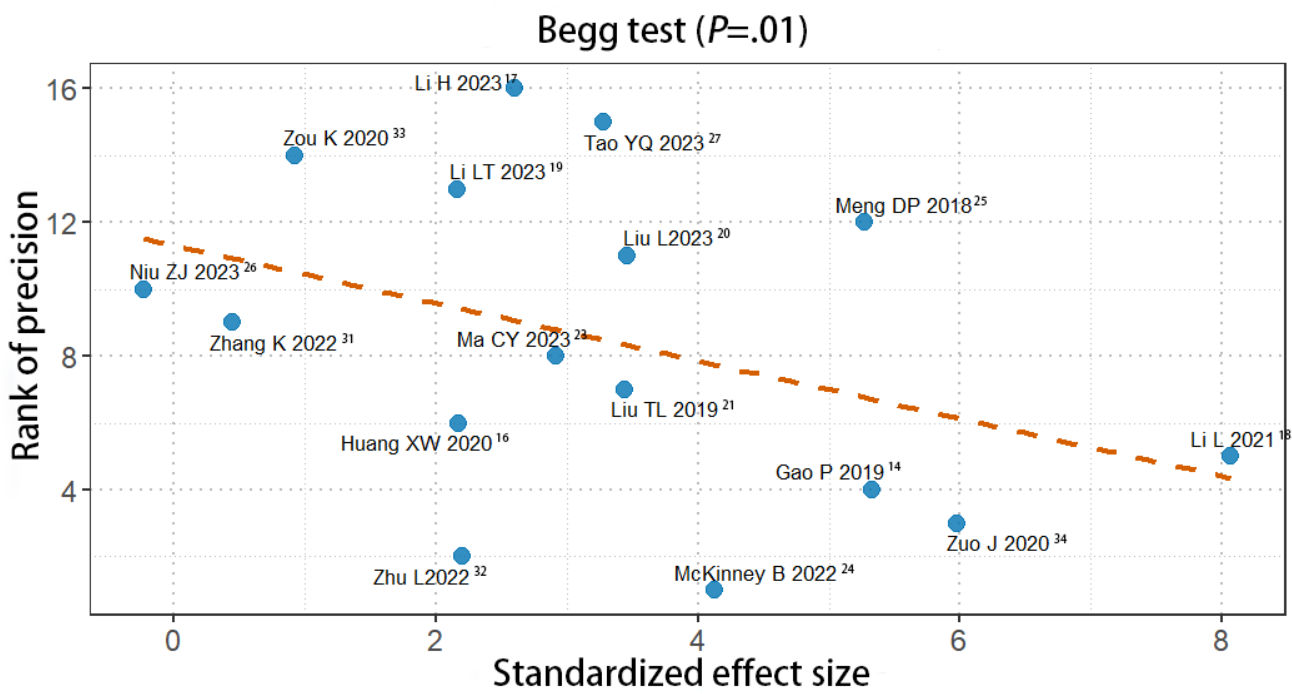
A Begg plot showing publication bias for knowledge scores [15,17-22,24-28,32-35].

**Figure 12.**
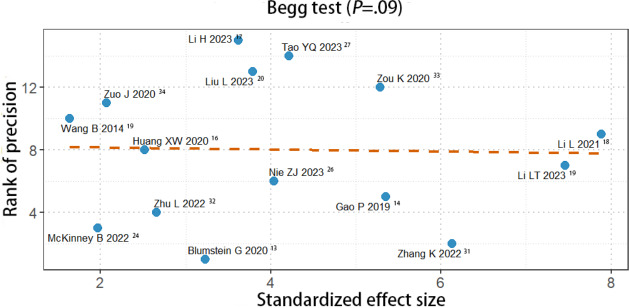
A Begg plot showing publication bias for clinical operation scores [13,15,17-21,25,27-29,32-35].

The Begg plot was used to evaluate the publication bias of studies. For studies in knowledge scores, the funnel plot had no symmetry (*P*=.01; see Figure 11). But, for studies in clinical operation scores, the funnel plot had symmetry (*P*=.09; see Figure 12) There is possibility of publication bias. However, we also detected publication bias in clinical understanding ability (see Figure S10 in Multimedia Appendix 3), which did not find the publication bias (*P*=.29).

## Discussion

### Principal Findings

This meta-analysis systematically reviewed 23 RCTs involving 1091 participants to evaluate the impact of VR teaching on orthopedic education compared to traditional teaching methods. The principal findings demonstrate that, compared to traditional teaching, VR interventions resulted in significantly higher scores in knowledge scores, clinical operation scores, and surgical design scores. Furthermore, VR-based instruction demonstrated enhanced clinical understanding and clinical thinking ability. Ultimately, this immersive methodology was shown to increase learner motivation while concurrently elevating student teaching interest and instructional satisfaction. Although the majority of included studies were RCTs, significant heterogeneity was observed across investigations, potentially attributable to variations in orthopedic surgical protocols, participant demographics, VR instructional durations, and outcome assessment metrics, collectively contributing to the elevated heterogeneity. Nevertheless, the synthesized results compellingly demonstrate the efficacy of VR-based interventions in enhancing orthopedic surgical education.

### Advantages of VR in Orthopedic Education

VR teaching is increasingly recognized as a promising innovation in medical education, particularly in the field of orthopedic surgery [36,37]. Orthopedics presents significant challenges due to its broad scope, encompassing trauma, sports injuries, joint conditions, and bone tumors. These subjects are intricate and interconnected with disciplines like anatomy, radiology, and biomechanics, posing difficulties in understanding and retention [38]. Consequently, VR teaching methods are widely embraced in orthopedic education to address these complexities. The integration of VR into orthopedic education has demonstrated several significant advantages over traditional teaching approaches. First, VR teaching enhances theoretical knowledge acquisition. The meta-analysis found a substantial SMD of 1.08 in knowledge scores favoring VR-based education compared to traditional teaching methods. This finding under-scores VR’s ability to provide a dynamic learning environment where learners can interact with and manipulate 3D models of anatomical structures and surgical procedures, leading to improved understanding and retention of complex concepts [39,40]. A meta-analysis found that 65 articles related to VR were categorized resulted in 45 pro the use of this technology, and this review highlights the important role of augmented reality and VR technology in anatomy curriculum [41]. Furthermore, VR teaching enhances practical skills and procedural competencies among orthopedic trainees. The higher clinical operation scores and surgical design scores highlight the effectiveness of VR simulations in facilitating hands-on practice and skill refinement. By allowing repeated and controlled simulations of surgical procedures, VR enables trainees to develop muscle memory, spatial awareness, and surgical dexterity in a safe and supportive environment [42,43]. This aspect is crucial for preparing surgeons to perform complex orthopedic surgeries with precision and confidence. In an RCT, 38 participants were allocated into two groups: a VR group (n=19) and a traditional teaching group (n=19). The VR group demonstrated significantly improved time to completion of surgical tasks compared to the traditional training group (*P*=.03) and exhibited fewer procedural errors (2.2 vs 2.5; *P*=.05). These findings indicate that VR-based training is more effective than traditional methods in facilitating learning and procedural execution among novice medical students during surgical procedures [44]. Therefore, VR teaching enhances the effectiveness and efficiency of orthopedic surgical education.

### Cognitive and Decision-Making Benefits

Beyond technical proficiency, VR improves critical thinking and clinical reasoning abilities. The meta-analysis revealed significant improvements in clinical understanding (SMD=1.05) and clinical thinking ability (SMD=1.17) with VR-based education. VR simulations offer realistic patient scenarios and diagnostic challenges that require learners to apply knowledge and make informed clinical decisions [45]. This active learning approach promotes problem-solving skills and prepares orthopedic surgeons to navigate clinical complexities encountered in real-world practice. However, A meta-analysis [46] of 12 RCTs found that no statistically significant impact was observed on the enhancement of critical thinking skills (SMD=0.79, 95% CI −0.05 to 1.64; *P*=.07) among nursing students. This may be due to different study results due to different participants included. On the contrary, other’s study described that VR teaching had the potential to enhance critical thinking [47]. Similarly, Cochrane et al [48] found that VR teaching can allow students to make an informed decision during the simulation. The term critical thinking in health has been synonymously aligned with clinical judgement, clinical reasoning, and decision-making [49]. Thus, this is consistent with our findings. Furthermore, VR teaching stimulates a proactive learning approach among students. The initiation of learning compared to traditional methods indicates that VR simulations foster curiosity, engagement, and self-directed learning. Interactive VR modules and case-based scenarios encourage learners to explore, experiment, and reflect on their practice, thereby enhancing motivation and knowledge acquisition [50]. VR teaching enables and encourages users to engage in interactive operations, which enhances learners’ attention and maintains high engagement throughout the interactive learning process. In addition, interactive operations are particularly effective in stimulating medical students’ self-directed learning capabilities. Thus, our meta-analysis found that VR-based teaching yielded significantly higher levels of initiative ability compared to traditional teaching methods.

### Educational Engagement and Satisfaction

The immersive nature of VR simulations enhances educational engagement and satisfaction among orthopedic trainees. Higher levels of teaching interest and teaching satisfaction associated with VR-based education reflect learners’ positive experiences with interactive learning environments. VR’s ability to provide immediate feedback, personalized learning experiences, and collaborative training opportunities contributes to a supportive and stimulating educational atmosphere [51]. However, a meta-analysis [52] found that there was no difference between VR teaching group and the control teaching group in satisfaction (95% CI −0.79 to 0.80; *P*=.99), confidence (95% CI −0.28 to 0.27; *P*=.99), which is contrary to our findings. Through analysis, it is found that this error may be caused by the difference between the implementation plan of the participants and the control group. On the contrary, other’s systematic review [53] identified a significant improvement in the VR group’s skill and satisfaction levels (95% CI 0.74-1.57; *P*<.001). Furthermore, both teachers and students reported high levels of ease of use and motivation for using VR. Not only will this increase students’ satisfaction and interest in teaching, but teachers will also feel more educational and clinical utility from VR simulations. Thus, studies have consistently shown that VR enhances learner motivation and commitment to learning [54].

### Challenges and Considerations

Despite the promising benefits of VR teaching in orthopedic education, several challenges and considerations merit attention. Variability in VR platforms, content quality, and instructional design across studies introduces heterogeneity that may affect the comparability and generalizability of findings. Obviously, the significant heterogeneity was found in most of the outcomes, and we speculated that source of the heterogeneity might be contributed by following reasons: (1) the experience and the proficiency were different among the teachers, medical students and medical trainee; (2) the standardization of examination and surgical procedure is not the same; (3) the risk bias of the VR device, as the quality of the VR may also lead to different educational outcomes; (4) furthermore, the baseline characteristics table of included studies revealed reporting gaps in participant age demographics and study duration parameters across several trials. Concurrently, methodological heterogeneity in both age distributions and intervention durations was observed, collectively introducing a potential risk of bias in the evidence synthesis. In the included studies, the sample sizes ranged from 18 to 125 participants. This variability primarily stems from differences in experimental design objectives (eg, small-sample exploratory trials vs large-sample confirmatory studies) and resource constraints. Notably, while all incorporated studies reported participant age and intervention duration in tabular formats, significant heterogeneity was observed in these variables (eg, age range: 21.16‐35.42 y and intervention duration: 3 d to 32 mo). Although the authors conducted sensitivity analyses, these failed to identify the sources of heterogeneity. In addition, publication bias assessments confirmed that a subset of studies exhibited publication bias. Standardization of VR teaching technologies and validation of educational outcomes through rigorous research methodologies, such as multicenter RCTs, are essential to establish evidence-based practices and guidelines for integrating VR into medical education [55]. Furthermore, future research should prioritize addressing identified gaps and advancing the application of VR in orthopedic education. Longitudinal studies are necessary to assess the sustained retention of knowledge and skills acquired through VR simulations and their impact on clinical practice outcomes. Comparative effectiveness research should investigate optimal VR training protocols, adaptive learning strategies, and personalized feedback mechanisms to maximize learning outcomes and enhance patient safety in orthopedic surgery [37,56]. Furthermore, the integration of emerging technologies, such as artificial intelligence and augmented reality, with VR platforms shows potential for creating more immersive, interactive, and tailored learning experiences in orthopedic education [57,58]. Besides, the cost of VR headsets and associated hardware (eg, computers, motion tracking devices, and specialized equipment) can be prohibitively high. VR technology requires ongoing maintenance, updates, and potentially software licensing renewals. These recurring costs can become a barrier for institutions with limited budgets, so addressing these challenges will require strategic investment, effective integration into curricula, and innovative solutions to make VR technology more affordable and accessible to a wider range of institutions. Finally, the lack of clear reporting on randomization in some studies and ambiguity in others raises concerns about the internal validity and reliability of the RCT design across the included studies. Continued exploration and innovation in these areas are crucial for harnessing the full educational potential of VR in orthopedic surgery.

### Limitations

However, there are also some limitations in our study: (1) first, the majority of included studies were randomized controlled trials; however, there was notable heterogeneity in terms of VR platforms, educational content, and outcome measures; (2) we only included studies reported in English and Chinese, which may have led to language bias, and this also might cause the source of heterogeneity; (3) long-term follow-up studies are needed to assess the sustainability of learning outcomes and the transferability of skills acquired through VR simulations to real-world clinical practice; (4) in addition, the cost-effectiveness of integrating VR into medical curricula remains a critical consideration for widespread implementation; and (5) some included studies did not report the age of participants or the detail of the study duration, which may lead to the bias of the outcomes. Hence, future research should aim to standardize these variables and conduct multicenter studies with larger sample sizes to further validate the efficacy of VR in orthopedic education.

### Conclusions

In summary, this meta-analysis supports VR as an effective tool in orthopedic surgery education, improving both knowledge and practical skills. Furthermore, it also markedly increasing student engagement and satisfaction. Therefore, adopting VR technology in medical education holds promise for improving orthopedic surgical competence. However, further validation through multicenter, double-blind, large-sample RCTs is necessary.

## Supplementary material

10.2196/70266 Multimedia Appendix 1 Index and keyword terms used in the databases.

10.2196/70266 Multimedia Appendix 2 GRADE (Grading of Recommendations Assessment, Development, and Evaluation) assessment of clinical outcomes.

10.2196/70266 Multimedia Appendix 3 Secondary meta-analysis results.

10.2196/70266 Checklist 1 PRISMA (Preferred Reporting Items for Systematic Reviews and Meta-Analyses) 2020 checklist.

## Abbreviations

AMSTAR: Assessing the Methodological Quality of Systematic Reviews
GRADE: Grading of Recommendations Assessment, Development, and Evaluation
MeSH: Medical Subject Headings
OR: odds ratio
PICOS: Population, Intervention, Comparison, Outcomes, and Study Design
PRISMA: Preferred Reporting Items for Systematic Reviews and Meta-Analyses
RCT: randomized controlled trial
SMD: standardized mean difference
VR: virtual reality
