# Supplementary material for: Using Virtual Reality to Enhance Surgical Skills and Engagement in Orthopedic Education: Systematic Review and Meta-Analysis
Source: J Med Internet Res. 2025 May 30;27:e70266. doi: 10.2196/70266 (PMC12143859; doi:10.2196/70266)
Supplement: Multimedia Appendix 1 [file jmir-v27-e70266-s001.docx]

Multimedia Appendix 1: Index and Keyword Terms Used in the Databases

| Databases | Search strategy |
| --- | --- |
| PubMed: | (("Orthopedics"[Mesh]) OR ((orthopedics[Title/Abstract]) OR (orthopedic education[Title/Abstract]))) AND (("Virtual Reality"[Mesh]) OR (((((((((((((((Reality, Virtual[Title/Abstract]) OR (Virtual Reality, Educational[Title/Abstract])) OR (Educational Virtual Realities[Title/Abstract])) OR (Educational Virtual Reality[Title/Abstract])) OR (Reality, Educational Virtual[Title/Abstract])) OR (Virtual Realities, Educational[Title/Abstract])) OR (Virtual Reality, Instructional[Title/Abstract])) OR (Instructional Virtual Realities[Title/Abstract])) OR (Instructional Virtual Reality[Title/Abstract])) OR (Realities, Instructional Virtual[Title/Abstract])) OR (Reality, Instructional Virtual[Title/Abstract])) OR (Virtual Realities, Instructional[Title/Abstract])) OR (patient simulat*[Title/Abstract])) OR (virtual patient*[Title/Abstract])) OR (virtual simulation[Title/Abstract]))) |
| Cochrane database: | #1 MeSH descriptor: [Virtual Reality] explode all trees  #2 (Reality, Virtual):ti,ab,kw OR (Virtual Reality, Educational):ti,ab,kw OR (Educational Virtual Realities):ti,ab,kw OR (Educational Virtual Reality):ti,ab,kw OR (Reality, Educational Virtual):ti,ab,kw  #3 (Virtual Realities, Educational):ti,ab,kw OR (Virtual Reality, Instructional):ti,ab,kw OR (Instructional Virtual Realities):ti,ab,kw OR (Instructional Virtual Reality):ti,ab,kw OR (Realities, Instructional Virtual):ti,ab,kw 61  #4 (Reality, Instructional Virtual):ti,ab,kw OR (Virtual Realities, Instructional):ti,ab,kw OR (patient simulate):ti,ab,kw OR (virtual patient):ti,ab,kw OR (virtual simulation):ti,ab,kw  #5 #1 OR #2 OR #3 OR #4  #6 MeSH descriptor: [Orthopedics] explode all trees  #7 (orthopedics):ti,ab,kw OR (orthopedic education):ti,ab,kw  #8 #6 OR #7  #9 #5 AND #8 |
| Embase: | #7. #3 AND #6  #6. #4 OR #5  #5. 'orthopedic surgery'/exp OR 'orthopedic surgery':ti,ab,kw OR 'orthopedic education':ti,ab,kw  #4. orthopedic OR 'orthopedic surgery'/exp  #3. #1 OR #2  #2. 'virtual reality'/exp OR 'reality, virtual':ti,ab,kw OR 'virtual reality, educational':ti,ab,kw OR 'educational virtual realities':ti,ab,kw OR 'educational virtual reality':ti,ab,kw OR 'reality, educational virtual':ti,ab,kw OR 'virtual realities, educational':ti,ab,kw OR 'virtual reality, instructional':ti,ab,kw OR 'instructional virtual realities':ti,ab,kw OR 'instructional virtual reality':ti,ab,kw OR 'realities, instructional virtual':ti,ab,kw OR 'reality, instructional virtual':ti,ab,kw OR 'virtual realities, instructional':ti,ab,kw OR 'patient simulation':ti,ab,kw OR 'virtual patient':ti,ab,kw OR 'virtual simulation':ti,ab,kw OR 'patient simulator':ti,ab,kw  #1. 'Virtual reality'/exp |
| Web of Science: | 1: orthopedic (Topic) OR orthopedics (Topic) OR orthopedic education (Topic)  2: ((((((((((((((((TS=(virtual reality)) OR TS=(Reality, Virtual)) OR TS=(Virtual Reality, Educational)) OR TS=(Educational Virtual Realities)) OR TS=(Educational Virtual Reality)) OR TS=(Reality, Educational Virtual)) OR TS=(Virtual Realities, Educational)) OR TS=(Virtual Reality, Instructional)) OR TS=(Instructional Virtual Realities)) OR TS=(Instructional Virtual Reality)) OR TS=(Realities, Instructional Virtual)) OR TS=(Reality, Instructional Virtual)) OR TS=(Virtual Realities, Instructional)) OR TS=(patient simulation)) OR TS=(patient simulator)) OR TS=(virtual patient)) OR TS=(virtual simulation)  3: #1 AND #2 |
| Scopus: | ( ( TITLE-ABS-KEY ( orthopedic ) OR TITLE-ABS-KEY ( orthopedics ) OR TITLE-ABS-KEY ( orthopedic AND education ) OR TITLE-ABS-KEY ( orthopedic AND surgery ) ) ) AND ( ( TITLE-ABS-KEY ( virtual AND reality ) OR TITLE-ABS-KEY ( reality, AND virtual ) OR TITLE-ABS-KEY ( virtual AND reality, AND educational ) OR TITLE-ABS-KEY ( educational AND virtual AND realities ) OR TITLE-ABS-KEY ( educational AND virtual AND reality ) OR TITLE-ABS-KEY ( reality, AND educational AND virtual ) OR TITLE-ABS-KEY ( virtual AND realities, AND educational ) OR TITLE-ABS-KEY ( virtual AND reality, AND instructional ) OR TITLE-ABS-KEY ( instructional AND virtual AND realities ) OR TITLE-ABS-KEY ( instructional AND virtual AND reality ) OR TITLE-ABS-KEY ( realities, AND instructional AND virtual ) OR TITLE-ABS-KEY ( reality, AND instructional AND virtual ) OR TITLE-ABS-KEY ( virtual AND realities, AND instructional ) OR TITLE-ABS-KEY ( patient AND stimulator ) OR TITLE-ABS-KEY ( patient AND simulation ) OR TITLE-ABS-KEY ( virtual AND patient ) OR TITLE-ABS-KEY ( virtual AND simulation ) ) ) |
| CNKI | (SU="虚拟现实技术"OR"虚拟教学"OR"模拟") AND SU="骨科" |
| VIP | (M=虚拟现实技术OR虚拟教学OR模拟) AND M=骨科 |
| Wan Fang | 主题:( 虚拟现实技术OR 虚拟教学 OR 模拟) AND 主题:(骨科) |
