# Supplementary material for: Using Virtual Reality to Enhance Surgical Skills and Engagement in Orthopedic Education: Systematic Review and Meta-Analysis
Source: J Med Internet Res. 2025 May 30;27:e70266. doi: 10.2196/70266 (PMC12143859; doi:10.2196/70266)
Supplement: Multimedia Appendix 2 [file jmir-v27-e70266-s002.docx]

Multimedia Appendix 2: GRADE assessment of clinical outcomes

| Certainty assessment | | | | | | |  | No. of patients | |  | Effect | |  | Certainty |
| --- | --- | --- | --- | --- | --- | --- | --- | --- | --- | --- | --- | --- | --- | --- |
| No. of studies | Study design | Risk of bias | Inconsistency | Indirectness | Imprecision | Other considerations |  | VR | Traditional teaching |  | Relative (95% CI) | Absolute (95% CI) |  |  |
| Knowledge scores (N=16) | RCTs | Serious ^a^ | Very serious ^c^ | No serious | Very Serious ^e^ | No |  | 397 | 397 |  | SMD 1.08 (0.71 to 1.46) | |  | ⨁◯◯◯Very low |
| Clinical operation scores (N=15) | RCTs | Serious ^a^ | Very serious ^c^ | Not serious | Very Serious ^e^ | No |  | 352 | 348 |  | SMD 1.44 (1.07 to 1.81) | |  | ⨁◯◯◯Very low |
| Operative design scores (N=5) | RCTs | Serious ^a^ | Serious ^b^ | No serious | Very Serious ^e^ | No |  | 69 | 69 |  | SMD 1.75 (1.05 to 2.44) | |  | ⨁◯◯◯Very low |
| Clinical understanding ability (N=10) | RCTs | Serious ^a^ | Serious ^b^ | No serious | Very Serious ^e^ | No |  | 184 | 184 |  | SMD 1.05 (0.62 to 1.48) | |  | ⨁◯◯◯Very low |
| Clinical thinking ability (N=5) | RCTs | Serious ^a^ | Serious ^b^ | No serious | Very Serious ^e^ | No |  | 91 | 91 |  | SMD 1.17 (0.66 to 1.68) | |  | ⨁◯◯◯Very low |
| Teaching interest (N=4) | RCTs | Serious ^a^ | No serious | No serious | Serious ^d^ | No |  | 79/103 | 50/103 |  | OR 4.17 (2.16 to 8.04) | |  | ⨁⨁◯◯ Low |
| Teaching satisfaction (N=5) | RCTs | Serious ^a^ | No serious | No serious | Serious ^d^ | No |  | 83/95 | 60/95 |  | OR 4.13 (1.96 to 8.69) | |  | ⨁⨁◯◯ Low |
| Initiative ability (N=7) | RCTs | Serious ^a^ | No serious | No serious | No serious | No |  | 163 | 163 |  | SMD 1.15 (0.91 to 1.39) | |  | ⨁⨁⨁◯Moderate |
| Course participation (N=4) | RCTs | Serious ^a^ | Very serious ^c^ | No serious | Very Serious ^e^ | No |  | 93 | 83 |  | SMD 1.25 (0.27 to 2.22) | |  | ⨁◯◯◯Very low |
| Interactive ability (N=3) | RCTs | Serious ^a^ | No serious | No serious | Very Serious ^e^ | No |  | 41 | 41 |  | SMD 1.26 (0.78 to 1.74) | |  | ⨁◯◯◯Very low |
| Learning efficiency (N=5) | RCTs | Serious ^a^ | Very serious ^c^ | No serious | Very Serious ^e^ | No |  | 92 | 92 |  | SMD 1.55 (0.47 to 2.62) | |  | ⨁◯◯◯Very low |
| Enhance clinical ability (N=3) | RCTs | Serious ^a^ | No serious | No serious | Very Serious ^e^ | No |  | 57/63 | 41/63 |  | OR 5.13 (1.90 to 13.87) | |  | ⨁◯◯◯Very low |
| Novelty of teaching (N=3) | RCTs | Serious ^a^ | No serious | No serious | Very Serious ^e^ | No |  | 52 | 52 |  | SMD 0.91 (0.50 to 1.32) | |  | ⨁◯◯◯Very low |
| Self-study ability (N=3) | RCTs | Serious ^a^ | No serious | No serious | No serious | No |  | 50/85 | 35/85 |  | OR 2.46 (1.23 to 4.95) | |  | ⨁⨁⨁◯Moderate |
| Self-confidence (N=2) | RCTs | Serious ^a^ | No serious | No serious | Very Serious ^e^ | No |  | 41 | 41 |  | SMD 0.65 (0.20 to 1.10) | |  | ⨁◯◯◯Very low |
| Solve problem ability (N=2) | RCTs | Serious ^a^ | No serious | No serious | Very Serious ^e^ | No |  | 42/60 | 20/60 |  | OR 5.68 (2.44 to 13.22) | |  | ⨁◯◯◯Very low |
| Train time (N=2) | RCTs | Serious ^a^ | No serious | No serious | Very Serious ^e^ | No |  | 20 | 20 |  | SMD -1.14 (-1.82 to -0.46) | |  | ⨁◯◯◯Very low |

Notes:

CI: confidence interval; OR: odds ratio; SMD: standardized mean difference.

Explanations:

a > 50% of trials received “High” risk of bias ratings (≥1 out of 6 dimensions in the Cochrane Risk of Bias tool);

b I^2^ between > 50% and ≤75% points in either direction;

c I^2^ >75% points in either direction;

d 95% CI of an SMD extends between > 0.2 and ≤0.5 points in either direction, 95% CI of an OR extends between > 5.0 and ≤10.0 points in either direction

e 95% CI of an SMD extends >0.5 points in either direction, 95% CI of an OR extends >10.0 points in either direction;
