# Supplementary material for: Using Virtual Reality to Enhance Surgical Skills and Engagement in Orthopedic Education: Systematic Review and Meta-Analysis
Source: J Med Internet Res. 2025 May 30;27:e70266. doi: 10.2196/70266 (PMC12143859; doi:10.2196/70266)
Supplement: Multimedia Appendix 3 [file jmir-v27-e70266-s003.docx]

Secondary meta-analysis results

**Figure S1.** A forest plot showing the course participation.


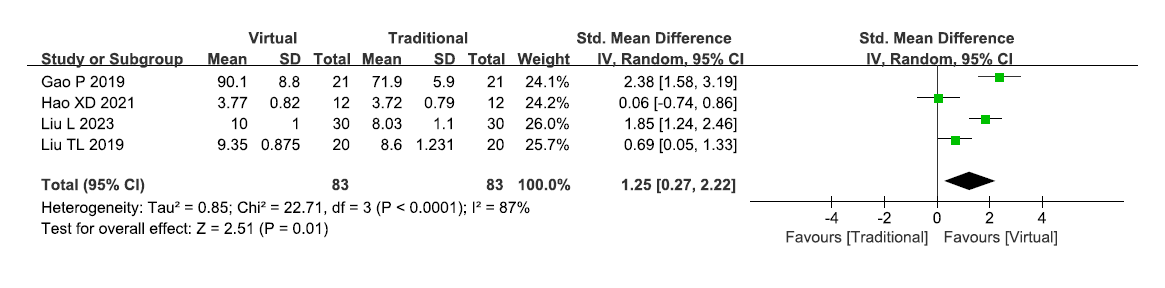


**Figure S2.** A forest plot showing the learning efficiency.


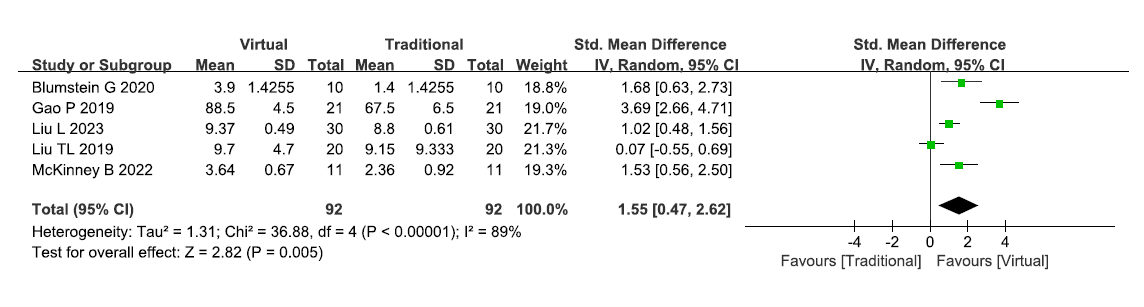


**Figure S3.** A forest plot showing the enhance clinical ability.


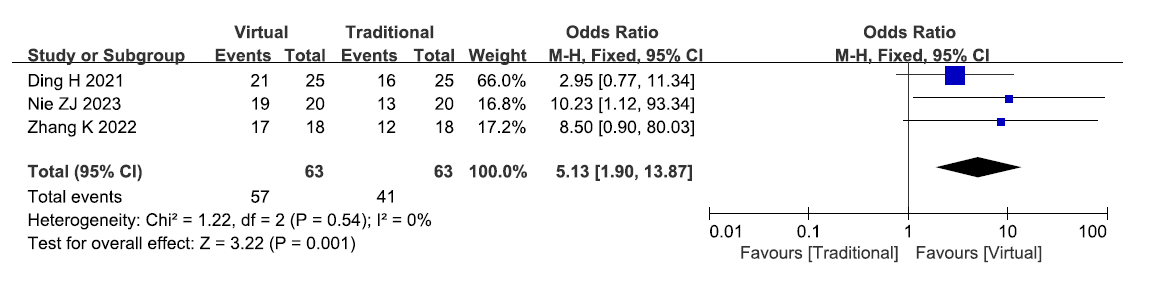


**Figure S4.** A forest plot showing the novelty of teaching.


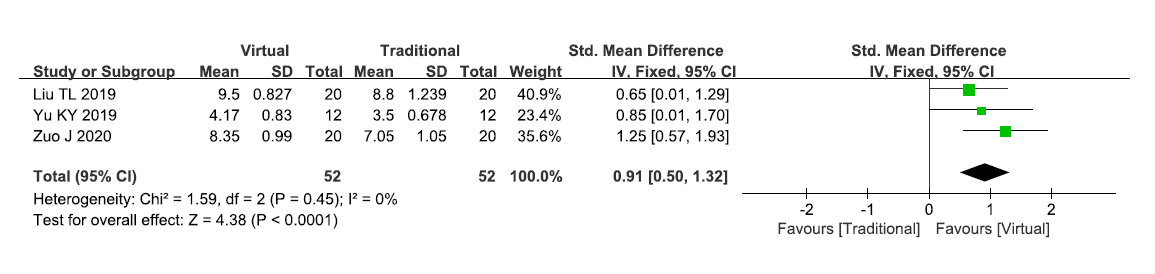


**Figure S5.** A forest plot showing the solve problem ability.


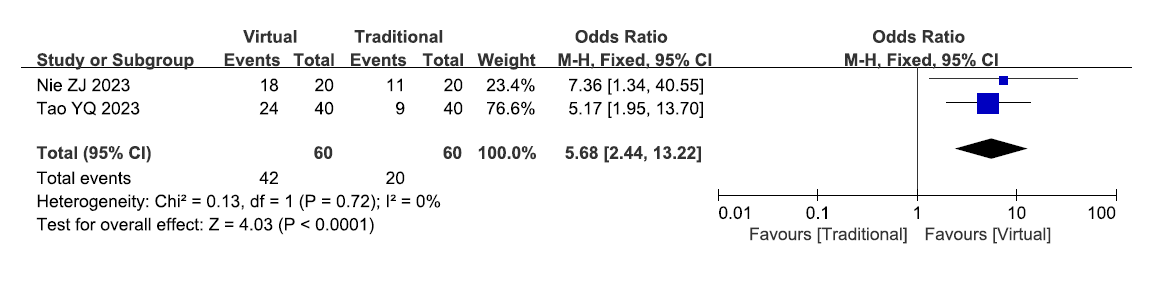


**Figure S6.** A forest plot showing the interactive ability.


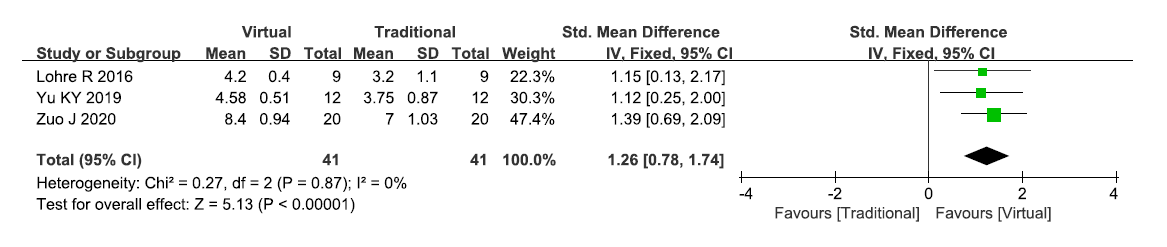


**Figure S7.** A forest plot showing the self-study ability.


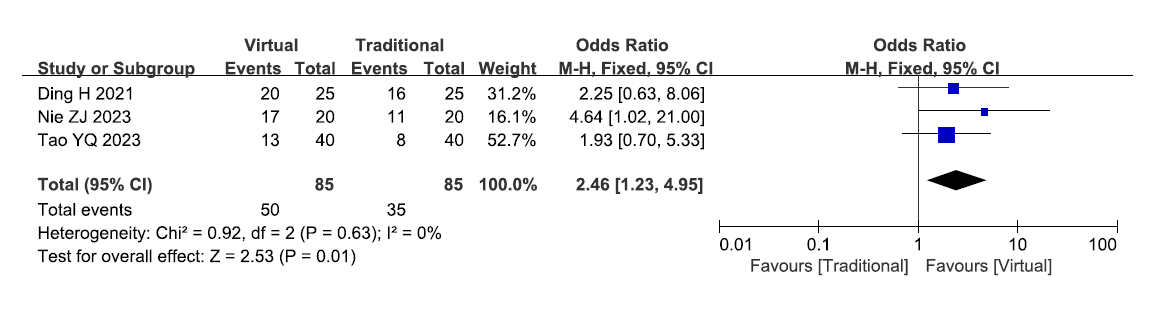


**Figure S8.** A forest plot showing the self-confidence.


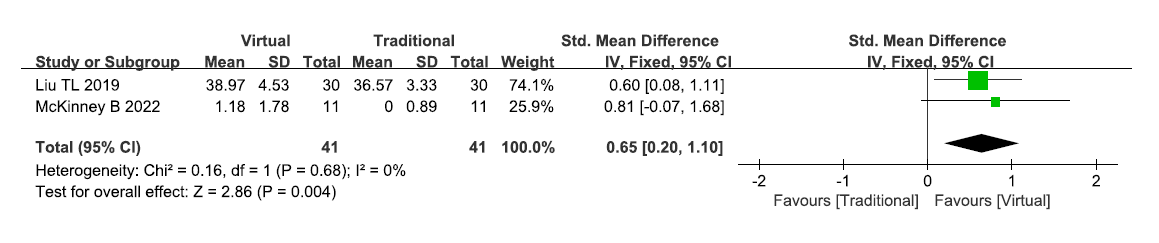


**Figure S9.** A forest plot showing the train time.


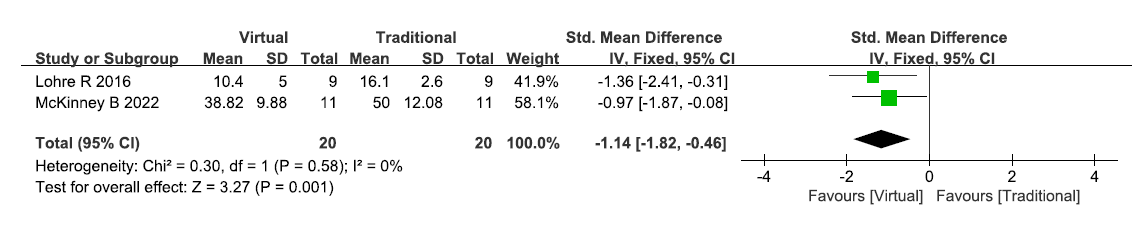


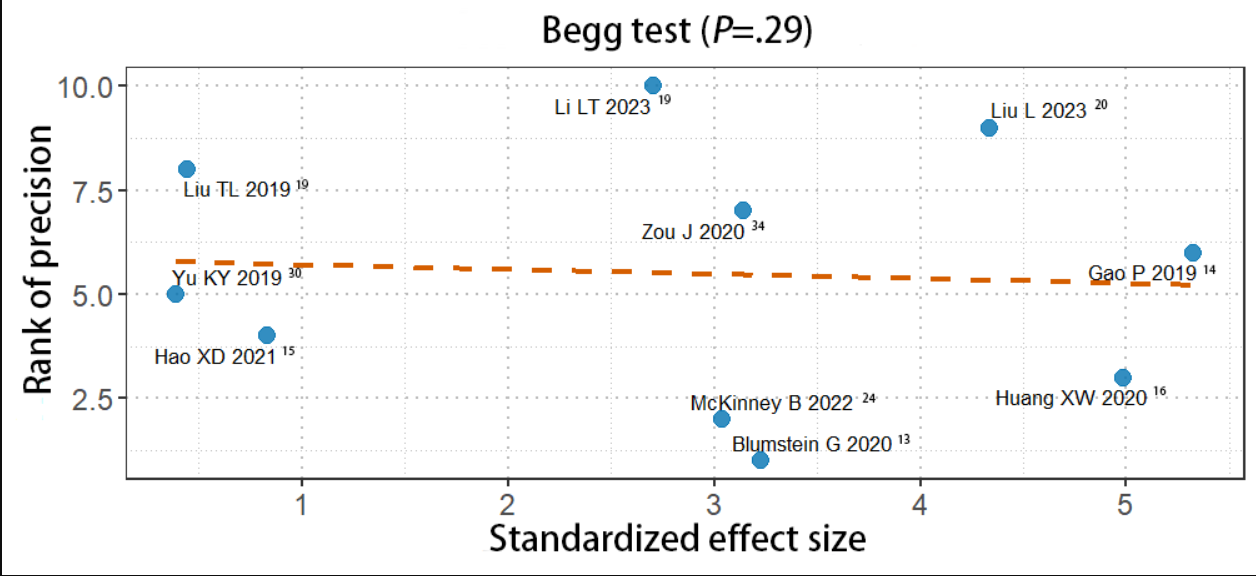
**Figure S10.** A Begg plot about clinical understanding ability.
